# Effect of methotrexate/dexamethasone combination on epithelial-mesenchymal transition and inflammation gene expression of human RPE cells *in-vitro*

**DOI:** 10.3389/fphar.2025.1569703

**Published:** 2025-04-30

**Authors:** Fatemeh Sanie-Jahromi, Mahdi Ravankhah, Hossein Shafi Khani, Seyed Ahmad Razavizadegan, M. Hossein Nowroozzadeh

**Affiliations:** Poostchi Ophthalmology Research Center, Department of Ophthalmology, School of Medicine, Shiraz University of Medical Sciences, Shiraz, Iran

**Keywords:** methotrexate, dexamethasone, apoptosis, inflammation, EMT, RPE, PVR

## Abstract

**Introduction:**

RPE cells serve as an experimental model for studying a retinal disease called proliferative vitreoretinopathy (PVR). The pathological background of PVR involves uncontrolled cell proliferation, increased inflammation, and enhanced epithelial-mesenchymal transition (EMT), which have been the focus of various research studies. The present study aimed to explore the effects of combination therapy using methotrexate (MTX) and dexamethasone (DEXA) on the expression of genes involved in apoptosis, inflammation and EMT in retinal pigment epithelial (RPE) cells.

**Methods:**

Our study design comprised two sets of experiments. First, we assessed the effect of MTX serial dilutions (0.5x, x, 2x, and 4x, where x = 100 μg/mL) on RPE cells to determine the optimal concentration of MTX that promotes apoptosis-related gene expression without altering inflammatory-related gene expression. Second, we investigated the influence of MTX (at the selected dose) alone or in combination with DEXA (50 μg/mL) on apoptosis, inflammation, and EMT-related gene expression in RPE cells at the transcriptional level.

**Results:**

Treatment with 100 μg/mL MTX demonstrated a pro-apoptotic effect according to the expression level of *BAX* and *BCL-2* in RPE cells. The combination of MTX (100 μg/mL) and DEXA significantly reduced the expression of inflammation-related genes (*IL-1b, IL-6*), indicating a synergistic anti-inflammatory effect. However, there was no significant effect on the expression of genes related to EMT (*TGF-β, CD90, β-Catenin, Snail*), except for a partial neutralization of the reducing effect of MTX on *ZEB1* and *α-SMA* genes.

**Discussion:**

Our study highlighted the potential pro-apoptotic effect of MTX (at 100 μg/mL) on RPE cells and the synergistic anti-inflammatory impact of MTX/DEXA combination therapy. Nevertheless, this combination did not significantly affect genes associated with EMT. Further research is required to elucidate the clinical implications of these findings in the management of PVR.

## 1 Introduction

Proliferative vitreoretinopathy (PVR) is a complex process characterized by the development of proliferative and contractile cellular membranes in the vitreous or above/below the retina, leading to tractional retinal elevation with fixed retinal folds. PVR represents a significant complication of rhegmatogenous retinal detachment (RRD) or its surgical repair, often resulting in unsuccessful procedures and subsequent vision loss (Ryan, 1985; Abrams and Glazer, 1993).

While the precise pathophysiology of PVR remains incompletely understood, its development involves a complex interplay of cellular and humoral factors. Notably, retinal pigment epithelial cells (RPE) play a pivotal role in PVR development (Tosi et al., 2014; Rodrigues et al., 1981; Ishikawa et al., 2015). These cells experience a loss of polarity and undergo epithelial-mesenchymal transition (EMT), marked by a shift from their typical epithelial morphology to a mesenchymal-like phenotype. In addition, inflammatory cytokines and chemokines contribute significantly to the pathogenesis of PVR. The transformed cells within the proliferative membranes transform into fibroblast-like cells containing actin and myosin, capable of contracting (Xu et al., 2019; Lei et al., 2011). Furthermore, apoptosis is another mechanism involved in the pathophysiology of PVR. Research has indicated that BCL-2 as an inhibitor of necroptosis pathway might be a novel target in PVR management (Li-Ping et al., 2005; Pastor-Idoate et al., 2015). These alterations (in apoptosis, inflammation and EMT) collectively result in a decrease in the retina’s elasticity, ending up in tractional elevation of the retina, the formation of new retinal tears, and recurrent retinal detachment (Khan et al., 2015).

Methotrexate (MTX) is valuable in managing PVR due to its dual action as an anti-proliferative agent and an anti-inflammatory modulator (Sadaka et al., 2016). By reducing intracellular folic acid levels, MTX affects purine and pyrimidine metabolism, leading to anti-proliferative effects (Surge in ophthalmic surgery predicted, 1986). Recent research also highlights its role in elevating extracellular adenosine levels, contributing to its anti-inflammatory properties (Surge in ophthalmic surgery predicted, 1986). However, understanding MTX’s impact on gene expression within this pathway at the mRNA level remains crucial. Additionally, dexamethasone (DEXA), as a commonly used therapy for inflammation-associated ocular diseases (Saraiya and Goldstein, 2011), lacks comprehensive studies when combined with MTX. Our study aims to explore the effects of MTX/DEXA combination therapy on RPE cell gene expression in an *in-vitro* setting.

## 2 Methods

### 2.1 RPE cell culture

The human eye globes were obtained from two male cadavers, aged 25 and 32 years, who had given their consent for organ donation. These globes were sourced from the Central Eye Bank of Iran and transported to the laboratory under sterile conditions. It is worth noting that globes intended for RPE explant culture must be utilized within 48 h after the donor’s passing. The initial step involved a thorough cleansing of the globes to remove any surrounding tissues. Following this, a small incision was made in the pars plana to evacuate the vitreous. Subsequently, the globe was sectioned from the periphery and opened up to gain access to the internal tissues. After rinsing the globe with sterile PBS, the neural retina was carefully extracted, leaving behind the pigmented layer, which represents the RPE cells, and was detached from the globe. The pigmented RPE monolayer was then cut into approximately 2 × 2 mm pieces and seeded as explant cultures onto the plate surface. To support the growth of RPE cells, a complete culture medium was prepared, consisting of DMEM/F12, fetal bovine serum (10% FBS), penicillin (120 g/mL), and streptomycin (220 g/mL). This medium was added to the explants. Subsequently, the plate was incubated at 37°C within a CO2 incubator (Afarid et al., 2023; Sanie-Jahromi et al., 2021). Approximately 10 days after initiating the culture, RPE cells began to grow from the periphery of the explants and necessitated weekly passaging.

To confirm and identify the RPE cells, the expression level of RPE65 was assessed in both the first and sixth passages. PCR analysis was conducted using 100 ng of RNA extracted from each passage, followed by gel electrophoresis (Nowroozzadeh et al., 2023). This method identifies the RPE cells by confirming the decreasing expression of RPE65 gene in repeated cultures. RPE cells from passages 5-7 were used for MTX and/or DEXA treatment, as well as subsequent analysis.

### 2.2 MTX/DEXA preparation, and study design

MTX (Trexoma^®^) was procured from Nanoalvand Co. (Iran), while DEXA was obtained from Darou Pakhsh Co. (Iran). Previous studies have reported a clinical concentration range of 0.5–1.5 mg for intravitreal MTX injections. Considering an approximate vitreous volume of 4 mL, this corresponds to concentrations between 125 and 375 μg/mL. In our study, we selected an arbitrary concentration denoted as ‘x’ (100 μg/mL) to investigate the effects of MTX in two sets of experiments.

In the first set, we examined the effect of MTX serial dilutions on RPE cells over a 24-h period. The tested concentrations included 0 (control), 50, 100, 200, and 400 μg/mL. We assessed apoptotic and inflammatory gene expression during this time.

In the second set, we evaluated the effect of MTX treatment (using the concentration determined in the first set) either alone or in combination with DEXA. We focused on genes related to apoptosis, inflammation, and EMT in RPE cells using real-time PCR. DEXA was administered at a concentration of 50 μg/mL (equivalent to 200 μg/0.05 mL of intravitreal injection, diluted in a 4 mL vitreous). The second set included the following groups: MTX, DEXA, MTX/DEXA combination, and a control group with no drug treatment.

**FIGURE 1:**
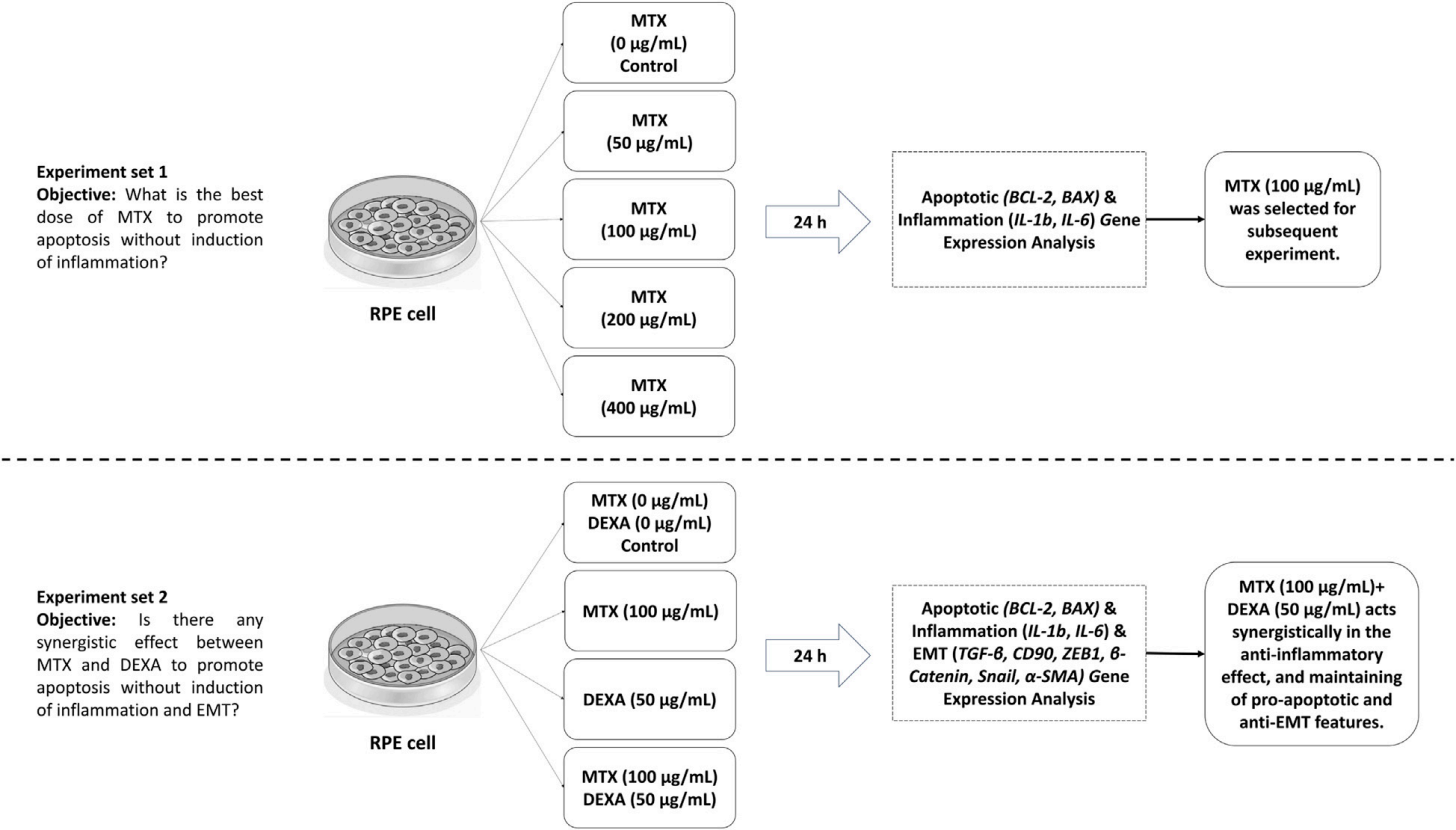
Study design and workflow.

Figure 1 represents the workflow of our study.

### 2.3 RNA extraction, cDNA synthesis, and gene expression analysis

RPE cells from passages 5-7 were seeded at a concentration of 1 × 10^6^ cells per 10 cm^2^ culture plate and treated with MTX and/or DEXA for 24 h, as described in the study design. Control groups consisted of RPE cells that did not receive any MTX/DEXA treatment. All treated and control cultures were prepared in triplicate. Total RNA extraction was performed using the RNeasy kit (Parstous) following the recommended protocol. cDNA synthesis was carried out using the Easy cDNA Synthesis Kit RNA (Parstous). The primer sequences for *BAX*, *BCL-2*, *IL-1b*, *IL-6*, *TGF-β*, *CD90*, *ZEB1*, *β-Catenin*, *Snail*, *α-SMA*, and *β-actin* (as internal control) were designed using the open-source NCBI primer designing tool (https://www.ncbi.nlm.nih.gov/tools/primer-blast) and their efficiency rates were verified using AllelID software (v.7.5, Premier Biosoft International, Palo Alto, CA, United States). Table 1 presents the primer sequences utilized in this study. Real-time polymerase chain reaction (PCR) was performed on the StepOneTM Real-Time PCR system (Applied Biosystems) to quantify the relative expression of the genes. Each PCR reaction had a volume of 15 μL, including 7.5 μL RealQ Plus Master Mix Green (Ampliqon), 1.5 μL of forward and reverse primers, and 6 μL of the target cDNA. The PCR thermal profile consisted of a 15-min hold phase at 95°C, a 10-s denaturation phase at 95°C, and a 45-s annealing and extension phase at 61°C. This cycle was repeated for 40 cycles. Finally, the 2^−ΔΔCt^ method was employed to evaluate the relative expression of the genes of interest.

**TABLE 1:** The primer sequences of the genes under study.

Gene	Sense primer	Anti-sense primer	Product size
BCL-2	CCC​GCG​ACT​CCT​GAT​TCA​TT	CAG​TCT​ACT​TCC​TCT​GTG​ATG​TTG​T	167 bp
BAX	TTC​TGA​CGG​CAA​CTT​CAA​CTG​G	CACAGGGCCTTGAGCACC	78 bp
IL-1b	AGC​AAC​AAG​TGG​TGT​TCT​CC	TGG​GAT​CTA​CAC​TCT​CCA​GC	153 bp
IL-6	TCCTTCTCCACAAGCGCC	ATG​CCG​TCG​AGG​ATG​TAC​C	185 bp
TGF-β	GCA​ACA​ATT​CCT​GGC​GAT​ACC	CCT​CAA​TTT​CCC​CTC​CAC​GG	123 bp
CD90	CTT​CAC​TAG​CAA​GGA​CGA​GGG	ACC​AGT​TTG​TCT​CTG​AGC​ACT	105 bp
ZEB1	CGC​AGT​CTG​GGT​GTA​ATC​GT	TTC​TTG​GTC​GCC​CAT​TCA​CA	216 bp
β-Catenin	CCG​AAT​GTC​TGA​GGA​CAA​GCC	TCA​AGT​CCA​AGA​TCA​GCA​GTC​TCA	117 bp
Snail	TAG​CGA​GTG​GTT​CTT​CTG​CG	CTG​CTG​GAA​GGT​AAA​CTC​TGG​AT	160 bp
α-SMA	CAC​GAT​GTA​CCC​TGG​GAT​CG	GCGGGGCGATGATCTTGA	89 bp
β-ACT	GCCTCGCCTTTGCCGAT	CATGCCGGAGCCGTTGT	98 bp

### 2.4 Statistical analysis

The experiments were performed in triplicate, and the obtained data were analyzed using one-way analysis of variance (ANOVA) and *post hoc* LSD test using the SPSS software (version 22, SPSS Inc., Chicago, IL, United States). A P < 0.05 was used to determine the statistical significance.

## 3 Results

### 3.1 RPE cell morphology and characterization

**FIGURE 2:**
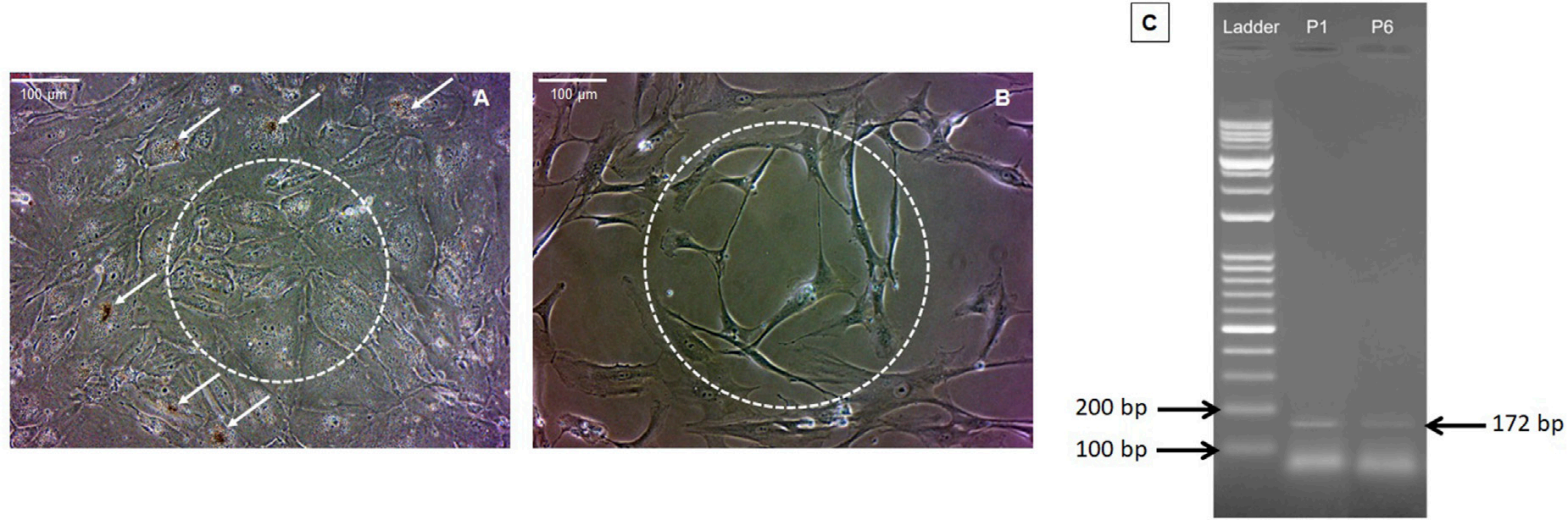
Morphological features of RPE cells. Panels **(A,B)** present phase-contrast images of RPE cells from passages 1 and 6, respectively (magnification ×20). In the first passage, RPE cells exhibited high pigmentation (indicated by arrows) and intercellular connections (depicted by dashed circles) **(A)**. However, pigmentation and cell junctions diminished in subsequent passages **(B)**. Agarose gel electrophoresis of RPE65 RT-PCR products for RPE cells from the first and sixth passages revealed a decrease in the level of RPE65 expression in the sixth passage compared to the first passage (confirming the typic characteristics of RPE cells). To assess this, 100 ng of total RNA was used from each passage of RPE cells. P1 denotes RPE65 mRNA expression in RPE cells from the first passage, and P6 represents RPE65 mRNA expression in RPE cells from the sixth passage **(C)**.

RPE cell growth was observed at the edges of the retinal explant under a phase-contrast microscope around 2 weeks after the initiation of culture. In the primary passages, RPE cells exhibited characteristic morphology, with abundant pigmentation and intact intercellular connections (Figure 2A). However, as the cell passages progressed, the RPE cells gradually lost their pigmentation and cell-cell connections, and subsequently acquired a spindle-shaped morphology (Figure 2B). Notably, our data demonstrated an inverse correlation between the expression level of RPE65 and the passage number of RPE cells confirming the identity of RPE cells *in-vitro* (Figure 2C).

### 3.2 RPE gene expression

#### 3.2.1 Apoptosis-related genes expression (*BCL-2* and *BAX*)

**FIGURE 3:**
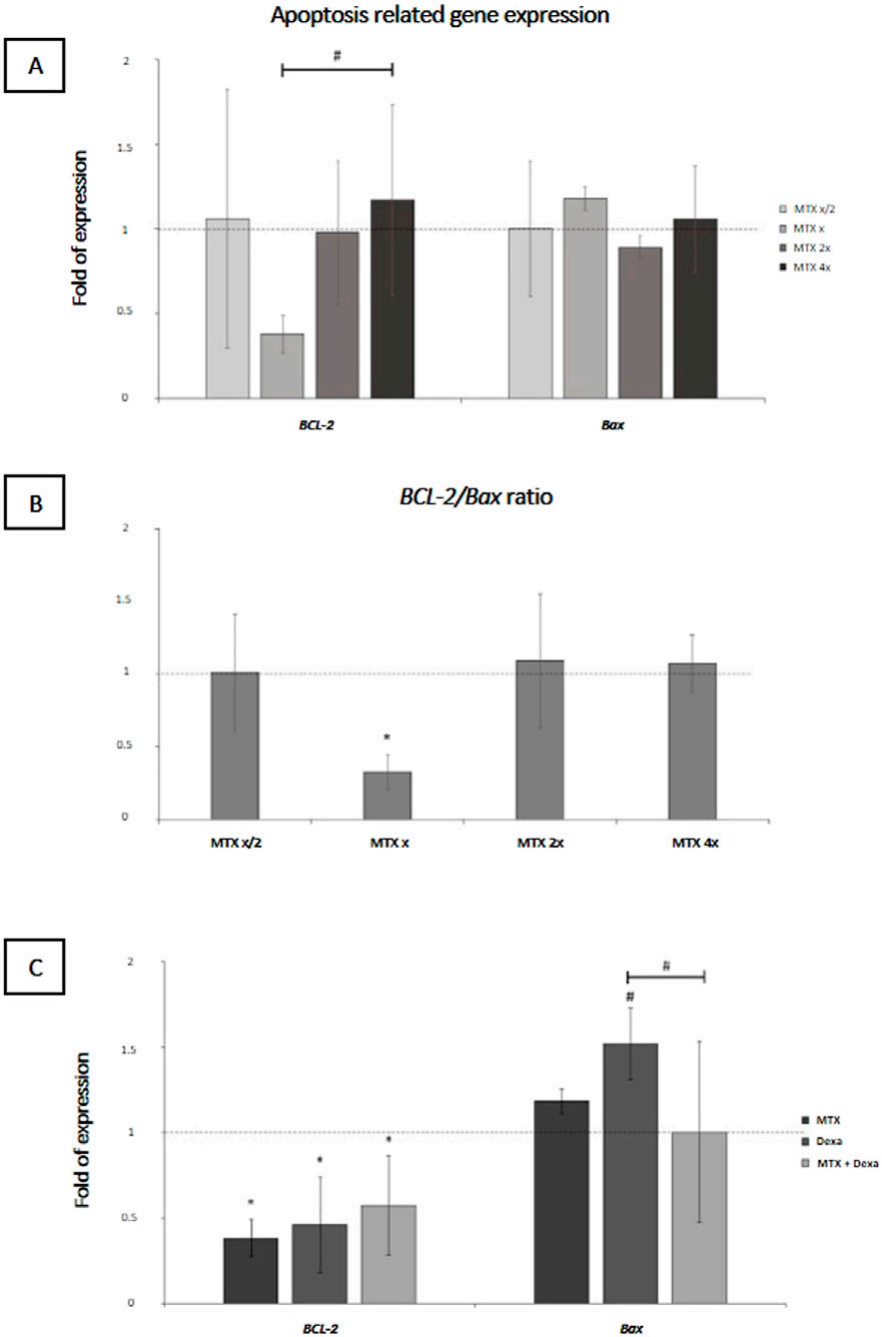
The apoptosis-related mRNA levels in RPE cells subjected to **(A)** serial dilutions of MTX (0.5x, x, 2x, and 4x, where x = 100 μg/mL) and **(C)** with MTX (100 μg/mL), DEXA (50 μg/mL), and combination of MTX + DEXA **(B)**. The dashed line represents the mRNA level in the controls (set as 1) following a 24-h treatment period. The graph presents the mean value of relative expression ± standard deviation of the mean. Statistical significance is denoted by * for P < 0.05 and # for P < 0.10, as determined by One-way ANOVA analysis (*post hoc* LSD, n = 3).

The effect of MTX at different concentrations is represented in Figure 3A. The mRNA expression of anti-apoptotic *BCL-2* showed a marginally significant downregulation at the concentration of 100 μg/mL MTX compared to 400 μg/mL concentration (0.38 ± 0.11, p-value = 0.063); moreover, the *BCL-2/BAX* ratio -at x concentration of MTX-was significantly lower than 1:1 (0.33 ± 0.12, P-value = 0.019), implying the pro-apoptotic effect of this dose on RPE cells (Figure 3B). Subsequently, we checked the mRNA level of *BCL-2* and *Bax* in MTX, DEXA, and MTX/DEXA treatments (Figure 3C) and showed that *BCL-2* expression was significantly reduced in all three treatments, and the *BCL-2/BAX* ratio was not significantly different between MTX, DEXA, and MTX/DEXA combination (all P > 0.1; data not shown).

#### 3.2.2 Inflammation-associated genes expression (*IL-1b*, *IL-6*)

**FIGURE 4:**
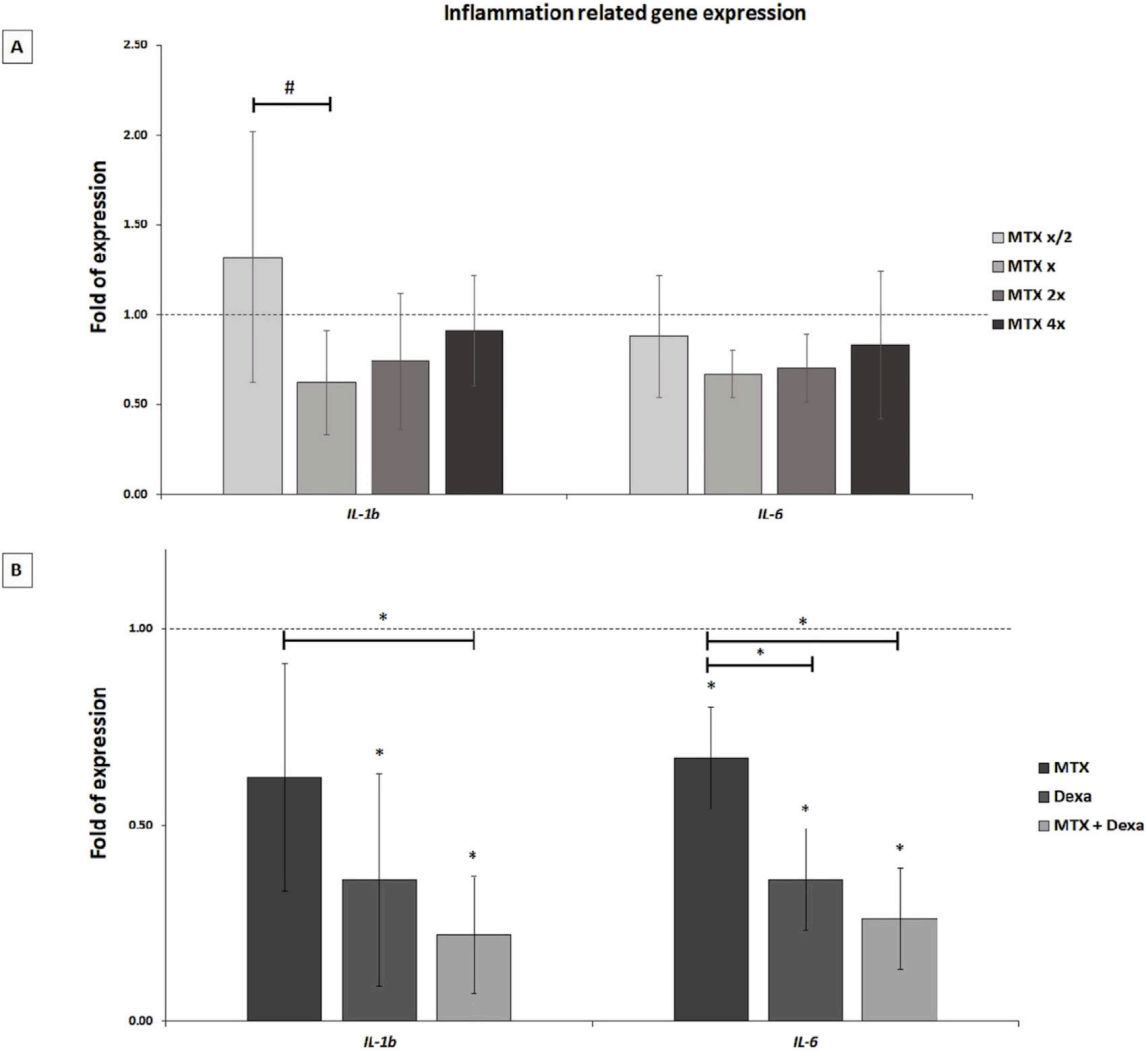
The inflammation-related mRNA levels in RPE cells exposed to **(A)** serial dilutions of MTX (0.5x, x, 2x, and 4x, where x = 100 μg/mL) and **(B)** MTX (100 μg/mL), DEXA (50 μg/mL), and combination of MTX + DEXA. The dashed line represents the mRNA level in the controls (set as 1) following a 24-h treatment period. The graph presents the mean value of relative expression ± standard deviation of the mean. Statistical significance is denoted by * for P < 0.05 and # for P < 0.10, as determined by One-way ANOVA analysis (*post hoc* LSD, n = 3).

The represented data demonstrated that treatment of RPEs with the x concentration of MTX did not lead to a significant alteration of *IL-1b* and *IL-6* gene expression in the 50, 100, 200, and 400 μg/mL MTX treated RPE cells compared to the control group (Figure 4A). Meanwhile, MTX/DEXA combination resulted in a significant decrease of *IL-1b* and *IL-6* gene expression, implying MTX and DEXA synergic effect on decreasing inflammation (Figure 4B).

#### 3.2.3 EMT-associated gene expression (*TGF-β, CD90, ZEB1, β-Catenin, Snail, α-SMA*)

**FIGURE 5:**
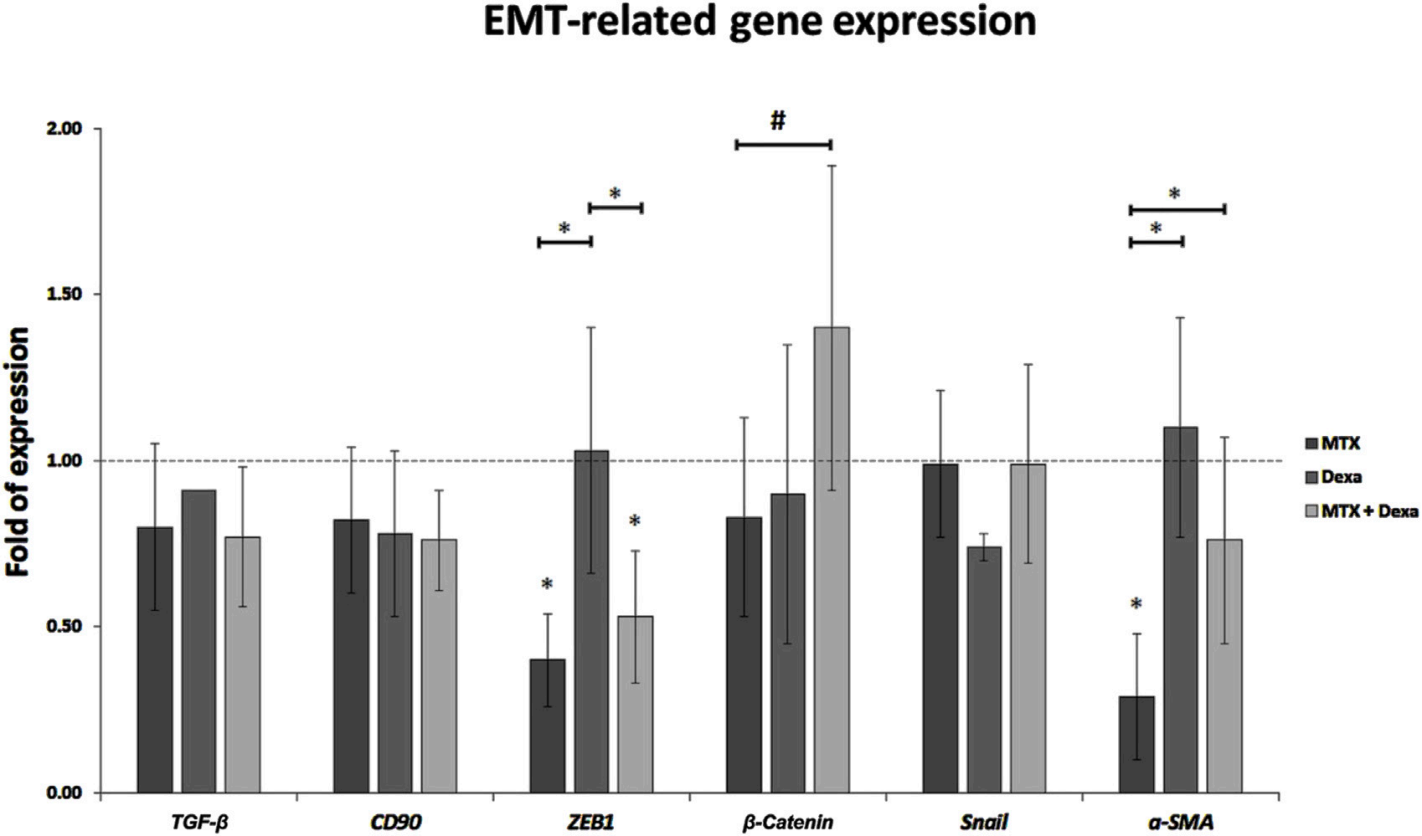
The relative expression of EMT-related genes in RPE cells treated with MTX (100 μg/mL), DEXA (50 μg/mL), and combination of MTX + DEXA. The dashed line represents the mRNA level in the controls (set as 1) following a 24-h treatment period. The graph presents the mean value of relative expression ± standard deviation of the mean. Statistical significance is denoted by * for P < 0.05 and # for P < 0.10, as determined by One-way ANOVA analysis (*post hoc* LSD, n = 3).

In this study, we evaluated the effect of MTX alone or in combination with DEXA on the mRNA expression of genes involved in the EMT process. We examined the expression of six important genes having a role in the EMT pathway. Based on the represented results, there was no significant difference in *TGF-β*, *CD90*, *β-Catenin*, and *Snail* between MTX, DEXA, MTX/DEXA combination, and control group. MTX/DEXA did not stimulate the expression of EMT-related genes. However, it could neutralize -to some extent-the reducing effect of MTX treatment on *ZEB1* and *α-SMA* gene expression (Figure 5).

## 4 Discussion

PVR is characterized by the uncontrolled proliferation of RPE cells, EMT, and inflammation (Datlibagi et al., 2023). It has been claimed that the therapeutic potential of MTX in managing PVR arises from its dual action as an anti-proliferative agent and an anti-inflammatory modulator (McAllister et al., 2023). Acting as a folic acid antagonist, MTX reduces intracellular folic acid levels, impacting purine and pyrimidine metabolism as well as amino acid synthesis, leading to anti-proliferative effects (Zarou et al., 2021). Recent studies have highlighted its role in elevating extracellular adenosine levels, adding an anti-inflammatory dimension to its profile (Riksen et al., 2006).

While the inhibitory effects of MTX on proliferation and inflammation, as a folic acid antagonist, have been confirmed, its impact on gene expression within this pathway at the mRNA level remains of significant importance. Considering the pivotal role of cytokines produced by RPE cells in the development of PVR, exploring MTX’s influence on inflammatory mediators and apoptosis responses in RPE cells can yield valuable insights for enhancing PVR management. By modulating inflammatory responses and promoting apoptosis at transcriptional level, MTX may play a pivotal role in mitigating the progression and severity of PVR.

Our study focused on the molecular aspects of MTX therapy on RPE cells, aiming to elucidate its potential applications for PVR management. Notably, the chosen *in-vitro* cell model closely mimics key features observed in PVR, making it a relevant platform for studying pathological processes associated with this condition. These features included a reduction in the expression of RPE65, loss of pigmentation, disruption of cell-cell contacts, undergoing EMT, acquiring a spindle-like morphology, and demonstrating a high proliferative potential (Gao et al., 2023).

Examining the impact of various MTX concentrations on apoptotic gene expression in RPE cells, we identified a distinct optimal concentration (x: 100 μg/mL, equivalent to 0.4 mg intravitreal injection) that significantly altered the *BCL-2/Bax* ratio, indicative of an appropriate induction of the mitochondrial apoptosis pathway. Furthermore, our investigation into the gene expression of *IL-1b* and *IL-6*, key inflammatory cytokines in PVR, demonstrated a reduction at the x concentration, though statistical significance was not reached.

Our findings suggest that the x concentration of MTX holds substantial transcriptional influence, manifesting proapoptotic and anti-inflammatory actions. In exploring potential synergistic effects, the combination of MTX and DEXA was investigated. While the combination did not alter the BCL-2/Bax ratio compared to MTX alone, it exhibited an enhanced reduction in *IL-1b* and *IL-6* mRNA expression, indicating a synergistic anti-inflammatory effect surpassing the individual impact of MTX.

This data is in line with previous investigations highlighting MTX’s efficacy in PVR treatment through promotion of apoptosis and inhibition of inflammation. Our laboratory data at the molecular level verifies positive clinical outcomes in terms of reattachment rates and visual improvement observed in human studies. In a study conducted by Benner et al., it was observed that all five patients who underwent intravitreal MTX treatment maintained retinal reattachment (100%) throughout a follow-up period ranging from 11 to 27 months (mean = 17.4). Among these patients, 4 eyes (80%) exhibited an improvement in ambulatory vision (>20/200), accompanied by normal intraocular pressure, non-fibrotic laser scars and relaxation retinectomy (Benner et al., 2019). Additionally, another study reported that adjuvant intravitreal MTX injections led to an enhancement of visual acuity to ≥20/200 in 19 out of 29 eyes (66%) with RRD/PVR after 6 months, with 24 out of 29 eyes (83%) showing either stability or improvement compared to their initial presentation (Sadaka et al., 2016).

Various studies have aimed to assess the efficacy of DEXA in PVR treatment, yielding controversial results. In a study conducted by Cho et al., six patients undergoing total vitrectomy, retinopexy, and silicone oil tamponade, with the addition of intraocular DEXA, were assessed. The investigation revealed improvements in visual acuity for two eyes, deterioration in one eye, and stability in four eyes. Notably, retinal attachment was sustained in all eyes throughout a 12-month follow-up period (Cho and Yoon, 2019). In another study, researchers concluded that the slow-release DEXA implant did not enhance the primary anatomic success rate in eyes undergoing vitrectomy surgery with silicone oil for PVR (Banerjee et al., 2017).

The combination of MTX and DEXA, as explored in our study, may offer enhanced efficacy in managing cell proliferation and reducing inflammation in PVR. Importantly, this combination does not exacerbate the EMT process, suggesting a favorable safety profile. *TGF-β, CD90, ZEB1, β-Catenin, Snail,* and *α-SMA* play central roles in RPE fibrogenesis and the EMT process (Li et al., 2020). Any therapeutic management that does not interrupt the expression of these genes might lead to a more suitable clinical outcome.

Currently, the primary management PVR involves the surgical removal of epiretinal and subretinal contractile membranes. Although this approach is successful in many patients, some still experience suboptimal anatomical or visual outcomes. Therefore, there is a need for effective medical strategies to prevent PVR, particularly in high-risk patients (e.g., pediatric retinal detachment, giant retinal tears, recurrent detachments, or detachments with stage ≥ B PVR), in order to improve the final outcomes of retinal detachment surgery in complex cases. Since PVR is widely regarded as an inflammatory and fibrotic complication, our findings on the synergistic effects of MTX combined with dexamethasone DEXA may have clinical implications for preventing PVR in high-risk patients and thereby increasing surgical success rates.

Despite these promising findings, it is essential to acknowledge the need for further in-depth studies, including *in-vivo* experiments and clinical trials, to validate our observations. The complexities of PVR warrant a comprehensive understanding of the molecular pathways involved in each therapeutic intervention, paving the way for more effective and targeted management of this challenging retinal condition.

## 5 Powers and limitations

This study is the first to investigate gene expression in RPE cells in response to varying concentrations of MTX and the MTX/DEXA combination, specifically targeting key genes involved in inflammation, apoptosis, and EMT—pathways critical to the development of PVR. By using primary human RPE cells, we aimed to better replicate the native physiological responses compared to immortalized cell lines. The consistent trends observed across two independent donors provide meaningful and reproducible insights, although expanding the donor pool in future studies would enhance generalizability.

While our *in-vitro* model offers a controlled environment to isolate the mechanistic effects of MTX/DEXA on RPE cells, it does not fully recapitulate the complex retinal microenvironment found *in-vivo*, including cell-cell interactions, systemic influences, and person-specific factors. Therefore, *in-vivo* validation in animal models remains an essential next step for translation. Moreover, although this study focused on transcriptomic changes as an initial screening approach, it did not explore protein-level interactions or pathway activations, nor did it include functional assays such as cell migration or invasion. These will be critical areas for future investigation.

In addition, potential off-target effects of MTX and DEXA—such as MTX’s impact on purine synthesis and DEXA’s non-genomic signaling—may influence RPE cell behavior independently of the EMT and inflammation pathways examined here. Finally, the predefined gene panel used may not capture all pathways modulated by MTX/DEXA, and broader transcriptomic or proteomic analyses are warranted in future studies to fully characterize their effects.

## 6 Conclusion

In summary, this study provided evidence supporting the pro-apoptotic, anti-inflammatory, and anti-EMT properties of MTX at a concentration of 100 μg/mL (approximately equivalent to 0.4 mg intravitreal injection). This concentration can serve as a reference for future *in-vivo* or clinical investigations. Additionally, the inclusion of DEXA at a concentration of 50 μg/mL (approximately equivalent to 200 μg/0.05 mL intravitreal injection) likely enhances the anti-inflammatory effects of MTX. Consequently, the combination of MTX and DEXA may be a viable option for preventing PVR in high-risk patients. Further studies are warranted to validate these findings and explore the clinical applications of this combination therapy.

## Data Availability

The data used to support the findings of this study are available from the corresponding author upon request.
